# Case report: Multi-site perfusion strategy for type A acute aortic dissection complicated with cerebral malperfusion

**DOI:** 10.3389/fcvm.2023.1124181

**Published:** 2023-03-06

**Authors:** Santiago Besa, Fiorenza Castelli, Luis Garrido-Olivares, Rodrigo González, Leopoldo Marine, Pedro Becker

**Affiliations:** ^1^Division of Surgery, Department of Cardiovascular Surgery, Pontificia Universidad Católica de Chile, Santiago, Chile; ^2^Medical School, Pontificia Universidad Católica de Chile, Santiago, Chile; ^3^Division of Surgery, Department of Vascular Surgery, Pontificia Universidad Católica de Chile, Santiago, Chile

**Keywords:** aortic arch surgery, aortic dissection, cerebral malperfusion, Frozen Elephant Trunk (FET), case report

## Abstract

Acute type A dissection presenting with cerebral malperfusion has high morbidity and mortality. Given the complexity of underlying vascular involvement, it is a challenging clinical scenario. Many of these patients are not deemed surgical candidates. If surgery is considered, it often requires complex aortic arch and neck vessel reconstruction. We present a 48-year-old male with an acute type A aortic dissection that presented with paraplegia and decreased level of consciousness. A Computed Tomography showed occlusion of both common carotid arteries. He was successfully treated with a multi-site perfusion strategy and a Hybrid Frozen Elephant Trunk graft to achieve fast restoration of the cerebral circulation and minimize brain ischemia and permanent neurological damage. From this case, we learn that aggressive arch and neck vessel reconstruction supported by multi-site perfusion could help improve mortality and neurological outcomes in selected patients.

## Introduction

Early mortality and morbidity after surgical treatment for acute type A aortic dissection (TAAD) have remained high over the last decades ranging between 10 and 30% (1). The patient's preoperative status remains the main predictor for outcomes after the surgery. Especially important is the presence of end-organ malperfusion, which is present in approximately one-third of patients (2). Cerebral malperfusion (CM) is the second most frequent form of malperfusion after the coronary arteries, and it is present in 5.2–13.1% of TAAD cases (3). A significant surgical dilemma is the treatment of TAAD patients with preoperative CM secondary to the involvement of supra-aortic branches, given its high mortality and risk for permanent neurological dysfunction.

The increasing availability of Hybrid Frozen Elephant Trunk (FET) prosthesis to address arch pathology, such as the Thoraflex (Terumo, FL, USA), gives a new perspective to treating TAAD involving the arch. Together with an aggressive and tailored cannulation strategy, they can help us treat this complex group of patients and achieve good outcomes (4).

We present a case of a TAAD involving all supra-aortic branches with severe neurological dysfunction where a multi-site perfusion strategy was used to perform emergent Thoraflex FET implantation. Fast and multi-site restoration of cerebral blood flow by this approach allowed to shorten brain ischemia time and minimize permanent neurological damage.

## Case report

**Figure 1:**
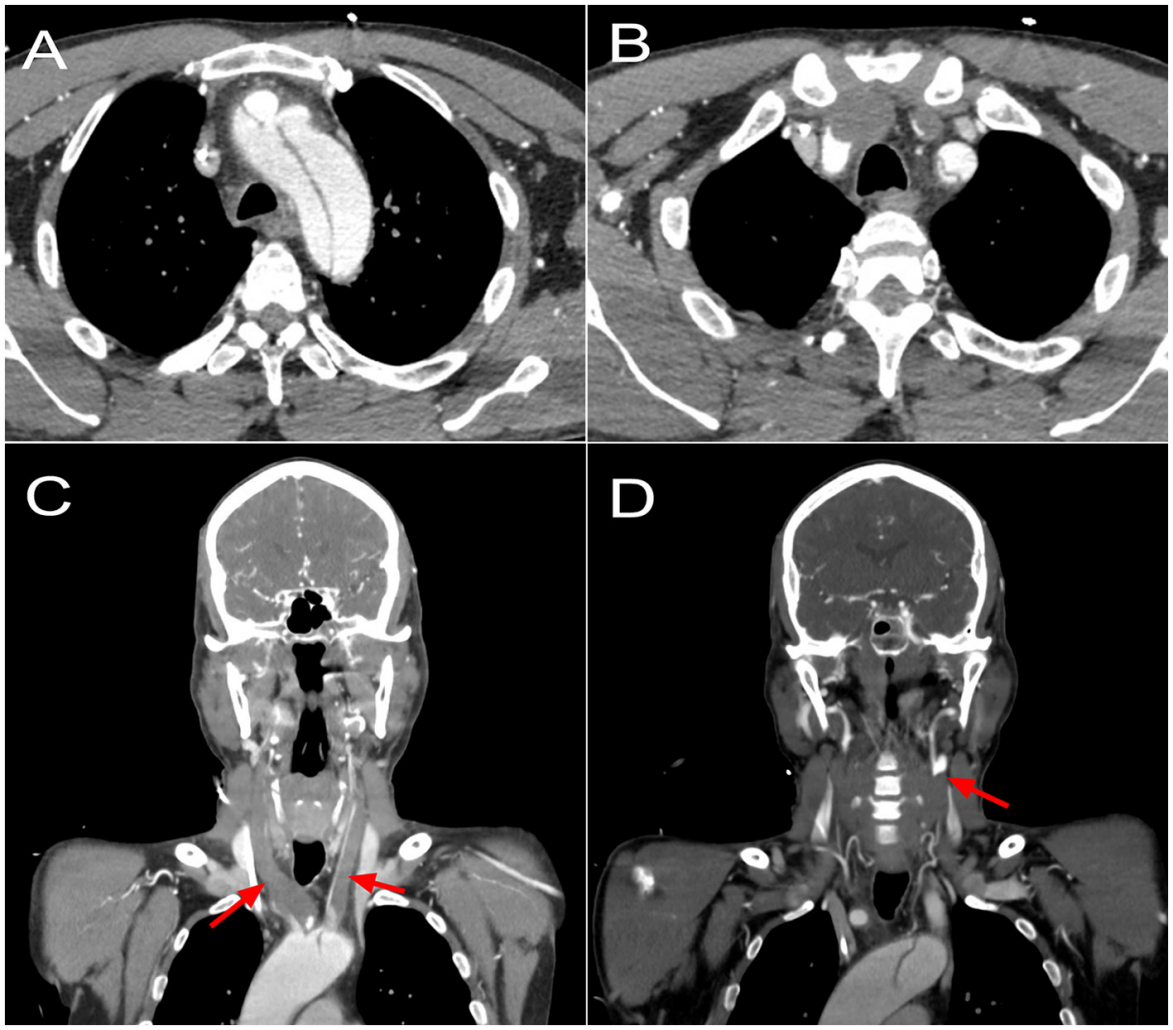
Preoperative CT scan showing **(A)** an acute type A dissection compromising the aortic arch and origin of supra-artic vessels. **(B)** A complete occlusion of the brachiocephalic trunk, with reperfusion of the right subclavian artery; a complete occlusion of the left common carotid artery; a dissected left subclavian artery. **(C)** A coronal reconstruction showing complete occlusion of both common carotid arteries since origin at the aortic arch (arrows). **(D)** A coronal reconstruction showing reperfusion at the level of the left carotid bifurcation (arrow).

A 48-year-old male with no known medical history and no family history of aortic disease or connective tissue disorders was admitted to a secondary Regional Hospital due to paresis of the lower right extremity. CT scan showed a TAAD starting at the proximal aortic arch and extending to the left internal iliac artery. All three supra-aortic vessels were compromised, with occlusion of both carotid arteries. The patient was transferred to our center for emergent surgery. The patient arrived at our center (1.5-h flight) in a deep stupor (Glasgow coma scale of 8) and with right hemiparesis (MRC strength scale of 1). A repeated CT showed a complete occlusion and thrombosis of the right common and internal carotid artery. The left common carotid artery was also occluded and thrombosed, showing recovered flow just before the bifurcation (Figure 1). The left subclavian artery was dissected, with involvement of the vertebral artery origin. There were no signs of consolidated cerebral infarction on CT. A GERAADA score (5) of 28.7% mortality at 30-day was calculated. Although its neurological prognosis was uncertain, there were no categorical elements of poor prognosis if cerebral circulation was restored. For this reason, we decided to proceed with emergency surgery.

**Figure 2:**
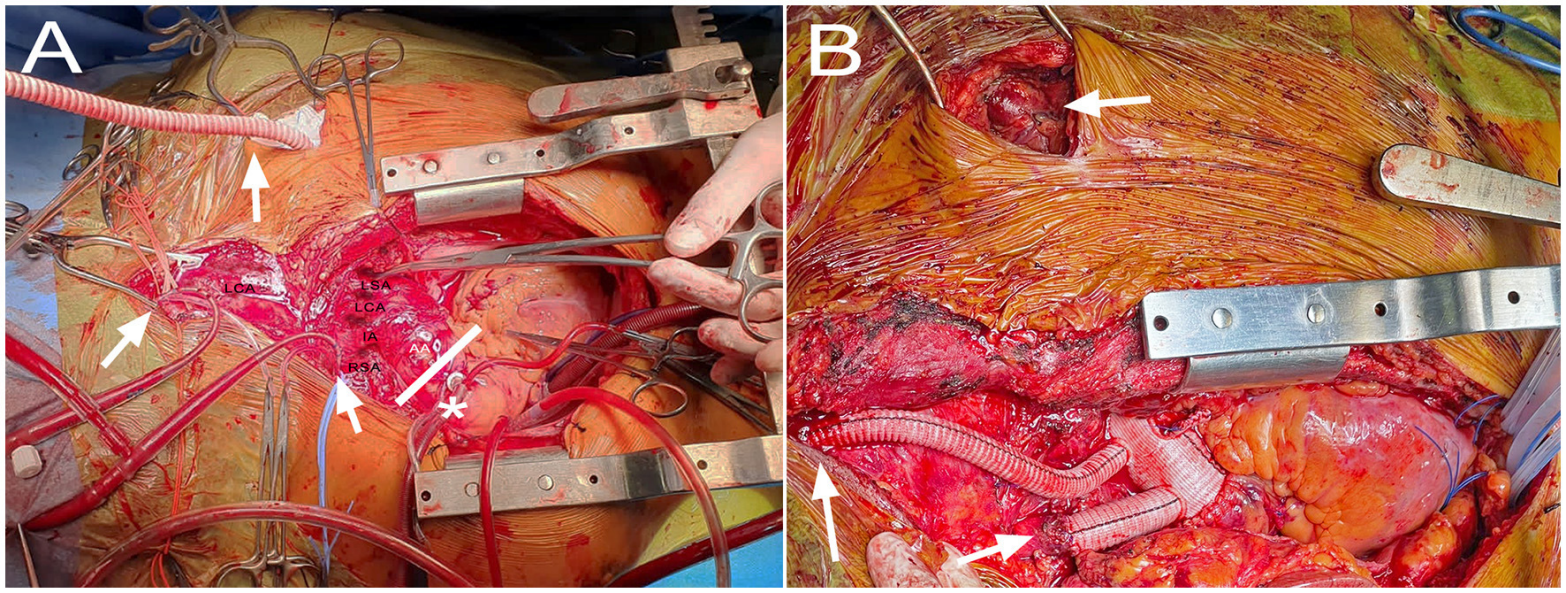
Intraoperative images showing **(A)** perfusion setup with direct ascending aorta (AA) cannulation in the uncompromised segment (*), pediatric cannulas to the right subclavian (RSA) and left internal carotid arteries, and Dacron graft to the left subclavian artery (arrows). The white lines depict the cross-clamp site to allow for cardiac perfusion through the aortic cannula. The innominate artery (IA), left carotid artery (LCA) and left subclavian artery (LSA) are also appreciated. **(B)** The final result with the Thoraflex deployed, and its branches anastomosed to the right subclavian artery, left carotid bifurcation, and left subclavian artery (arrows).

Bilateral radial and left femoral arterial lines were installed. Bilateral near-infrared spectroscopy (NIRS) was used. An incision from the left neck through the xiphoid was made. While the median sternotomy was performed, we controlled the left common, internal, and external carotid artery. The aorta was dissected from a short segment proximal to the innominate trunk, with a normal aspect of the root. All the supra-aortic vessels were compromised. There was no innominate vein, with a persistent left vena cava. We rapidly cannulated the left internal carotid artery with a 10-french pediatric aortic cannula to ensure cerebral perfusion. This cannula was perfused independently of the systemic line at 10 ml/kg throughout the procedure. We cannulated the ascending aorta in the uninvolved segment. Cardiopulmonary bypass (CPB) was initiated, inducing hypothermia to 24 °C. To achieve the best cerebral perfusion through the surgery, we decided to secure perfusion through the posterior territories by perfusing both subclavian arteries. The right subclavian artery was directly cannulated with a 12-french pediatric aortic cannula. The left subclavian artery was compromised in the observed segment, so we made a left sub-clavicular incision, and a Dracon graft was anastomosed in a terminal-lateral fashion. For the subclavian branches, we obtained flow from the systemic cannula (Figure 2).

The NIRS became adequate and symmetrical soon after CPB was initiated. We completed the dissection and control of all supra-aortic vessels. The left carotid artery and left subclavian artery were ligated at their origin. A guidewire from the right femoral artery was advanced through the true lumen with TEE support. We began systemic arrest. For this, an aortic clamp was installed distal to the aortic cannula, thereby maintaining pressure-regulated cardiac perfusion. In addition, we maintained the perfusion through the left carotid and both subclavian lines. The ascending aorta was opened, observing an intimal tear proximal to the innominate trunk. There was no retrograde flow through the right carotid artery. The arch was resected up to zone 2. A 30 × 32 × 100 mm Thoraflex hybrid prosthesis was deployed through the previously advanced guidewire and was anastomosed to the distal arch. The circulatory arrest was ended by starting the perfusion through the lateral branch of the Thoraflex graft. We then arrested the heart for a brief period to perform the proximal aorto-graft anastomosis in the mid-portion of the ascending aorta, after which the aortic cross-clamp time was completed. We continued with the supra-aortic vessel's anastomosis. The left subclavian graft was tunneled through the second intercostal space and anastomosed to its corresponding branch. The left carotid anastomosis was performed at the level of the carotid bifurcation (Figure 2).

Finally, we confirmed no retrograde flow in the right carotid artery, even after a Fogarty catheter was advanced. We excluded the right carotid, which was ligated, and performed the last branch anastomosis to the right subclavian artery. Once 36°C was reached, we weaned the patient off CPB without further incidents. The cross-clamp time was 21 mins and the systemic arrest time with antegrade cerebral perfusion was 28 mins.

On day 0, the patient had a cardiac arrest with ventricular fibrillation that required three shocks of defibrillation and recovered after 7 mins of resuscitation. An amiodarone infusion was started with no further events. He was weaned off vasoactive support on day 2. A head CT on day 2 showed small acute-subacute ischemic lesions on the left frontoparietal white matter. Weaning from mechanical ventilation was difficult because of severe delirium, requiring a tracheostomy on day 7. A new head CT on day 8 showed a new right cortico-subcortical frontal subacute infarction. He continue to improve clinically from a neurologic perspective, underwent neurological rehabilitation, and recovered complete mobility of his right side with minimum impairment (MRC strength scale of 4). He was decannulated from his tracheostomy on day 20. The patient was discharged on postoperative day 30 in good general condition.

## Comment

Despite surgical technical advances, operative mortality for TAAD remains high. There is a wide variety of presentations, and the preoperative status is still one of the main factors in predicting operative mortality after surgical repair. In particular, neurological deficits resulting from CM have been reported as a sign of poor prognosis (6). However, the optimal management for patients with CM is unclear. The last report of the International Registry of Acute Aortic Dissections in 2019 reported an incidence of CM in TAAD of 15.1% with an operative mortality of 25.7% (7). The mortality reported is encouraging in this highly complex clinical scenario but must be carefully interpreted. The reported case presents a highly tailored approach and highlights the importance of pre-surgical evaluation and assessment for cannulation sites and perfusion strategies.

There are many surgical techniques to cannulate patients with TAAD complicated by CM, most based on retrospective series or expert opinions. Still, there is no “one fits all” type of cannulation. Carotid artery cannulation has been advocated as a safe cannulation method for aortic surgery, mainly by P. Urbansky (8). The main advantages are that it is easy and fast (suitable for emergencies), offers adequate arterial return, and offers the possibility of early establishment of cerebral perfusion without interruption. It is an excellent alternative to the most frequently used axillar cannulation strategy, given that the innominate artery is frequently involved in dissection, possibly making it unsuitable for cannulation.

The present case was challenging because of the involvement of both common carotid arteries. Therefore, unconventional cannulation was done by cannulating the left internal carotid artery with a pediatric aortic cannula. Moreover, given the extensive preoperative compromise of anterior cerebral circulation, we decided to secure perfusion through the posterior territories by perfusing both subclavian arteries, achieving similar 4-site perfusion as proposed by Tsagakis et al. (4).

This case also shows that minimizing aortic cross-clamp time by maintaining isolated cardiac perfusion is possible in specific scenarios. Reducing cardiac ischemia time to a minimum is essential for a good cardiac outcome, and non-cardioplegic continuous myocardial perfusion has been shown to have lower cardiac mortality, lower rate of bleeding complications, and more effective myocardial protection (9).

The Hybrid FET technique offers an innovative approach to complex aortic arch repair operations and has revolutionized this field of surgery. The use of Thoraflex presents some advantages over other FET devices without supra-aortic branches, simplifying the reconstruction and minimizing surgical time.

In conclusion, the use of multi-site perfusion strategies and Hybrid FET grafts could be considered to treat patients presenting with TAAD and CM to minimize brain ischemia time, and facilitate the aortic arch reconstruction and cerebral blood flow restoration.

## Data availability statement

The original contributions presented in the study are included in the article/supplementary material, further inquiries can be directed to the corresponding author.

## Ethics statement

Written informed consent was obtained from the participant/patient(s) for the publication of this case report. Written informed consent was obtained from the individual(s) for the publication of any potentially identifiable images or data included in this article.

## Author contributions

SB, FC, and LG-O wrote the main manuscript. SB, RG, PB, and LM planned and performed the procedure. All authors reviewed the manuscript.
